# Increased Rates of *Purpureocillium lilacinum* Mold among Laboratory Culture Results, United States

**DOI:** 10.3201/eid3110.250715

**Published:** 2025-10

**Authors:** Dallas J. Smith, Luisa F. López, Meghan Lyman, Claire Paisley-Jones, Kaitlin Benedict

**Affiliations:** Centers for Disease Control and Prevention, Atlanta, Georgia, USA (D.J. Smith, L.F. López, M. Lyman, K. Benedict); US Department of Agriculture, Washington, DC, USA (C. Paisley-Jones)

**Keywords:** *Purpureocillium*, mold, hyalohyphomycosis, laboratories, fungi, United States

## Abstract

*Purpureocillium lilacinum*, a common environmental mold and bionematicide, can cause human infections. At a major US commercial laboratory during March 2019–February 2025, *P. lilacinum* culture rates increased; rates were highest in the South Atlantic and Pacific states. Nonculture-based diagnostic tools such as microscopy may help identify and confirm clinical infection earlier.

*Purpureocillium lilacinum* (formerly *Paecilomyces lilacinus*) is a naturally occurring filamentous fungus that is common in the environment, particularly in soil and decaying vegetation (*1*). Two strains of *P. lilacinum* registered in 2005 and 2021 are used as agricultural bionematicides in the United States (*2*,*3*). The fungus rarely causes human infection but can cause hyalohyphomycosis, an infection with varied clinical soft-tissue, ocular, or pulmonary manifestations. Infection most frequently affects immunocompromised persons but can also occur in immunocompetent persons. Mortality rates can reach 20% (*1*). 

*P. lilacinum* infection is clinically indistinguishable from other mold infections, and the organism resembles other molds on cytology, histopathology, and culture, potentially leading to misidentification and to delayed or inappropriate treatment (*1*). *P. lilacinum* is intrinsically resistant to amphotericin B and can be correlated with poorer treatment outcomes (*1*,*4*).

Worldwide, clinical characteristics and outcomes of 101 *P. lilacinum* infections have been described, 31 of which were from the United States, but epidemiology of the infection in the United States is poorly understood (*1*). We explored *P. lilacinum* culture data from a large national commercial laboratory to describe this organism in the United States.

We used data from the Centers for Disease Control and Prevention’s National Syndromic Surveillance Program (NSSP) (https://www.cdc.gov/nssp/index.html), which collects data from Labcorp (https://www.labcorp.com), a major national commercial laboratory network. Labcorp transmits test orders and results for all reportable diseases in the United States to NSSP. Although *P. lilacinum* infection is not reportable to public health authorities, NSSP receives data on all fungal cultures performed at Labcorp because other fungal diseases are reportable (*5*). We identified culture results for *P. lilacinum* ordered during March 1, 2019 (earliest available data) through February 28, 2025.

We examined demographic characteristics, geographic location of submitting provider’s state (US Census division), specialty or setting of the ordering healthcare provider, specimen type, and time from test order to culture result. Unique patient identifiers were unavailable; therefore, we conducted analyses at the test-result level.

We identified 1,180 *P. lilacinum* cultures (Table; Appendix Figure 1). Rates of *P. lilacinum* per 100,000 fungal cultures per year increased from 56.6 in 2019 to 74.3 in 2024 and peaked at >90 in the third quarter of 2024 (Figure). Overall, male persons (68.8/100,000 population) and persons >65 years of age (89.7/100,000 population) had the highest culture rates. By US Census division, the South Atlantic (110.6/100,000 population) and Pacific (100.3/100,000 population) divisions had the highest rates. Most (52%) *P. lilacinum* cultures were ordered from hospital settings. Specimen type was available for 57% of cultures, among which respiratory specimens were most common (38%). The median time from collection to result was 23 (interquartile range 15.0–33.0) days.

**Table:** Patient characteristics in study of increased rates of *Purpureocillium lilacinum* mold among laboratory culture results, United States*

Characteristic	Value	Rate†
Year of sample collection, n = 1,180		
2019	136 (12)	56.6
2020	134 (11)	50.5
2021	182 (15)	59.3
2022	224 (19)	71.7
2023	210 (18)	62.8
2024	258 (22)	74.3
2025	36 (3)	61.9
Median patient age, y (IQR)	65.0 (53.0–74.0)	
Age group, y, n = 1,175		
0–17	40 (3)	21.6
18–44	154 (13)	41.0
45–64	357 (30)	59.7
>65	624 (53)	89.7
Sex, n = 1,159		
M	607 (52)	68.8
F	552 (48)	58.9
US Census division, n = 1,172‡		
East North Central	91 (8)	69.1
East South Central	139 (12)	78.9
Middle Atlantic	11 (1)	3.6
Mountain	13 (1)	11.6
New England	<10 (<1)	28.7
Pacific	263 (22)	100.3
South Atlantic	603 (51)	110.6
West North Central	<10 (<1)	6.7
West South Central	44 (4)	17.3
Provider type, n = 1,100		
Hospital	567 (52)	
Family practice or internal medicine	132 (12)	
Dermatology	111 (10)	
Podiatry	71 (6)	
Pulmonary disease	55 (5)	
Otolaryngology	45 (4)	
Ophthalmology	14 (1)	
Other	105 (10)	
Median days from collection to result (IQR)	23.0 (15.0–33.0)	
Specimen type, n = 670		
Respiratory	254 (38)	
Nail	106 (16)	
Skin	82 (12)	
Sinonasal	13 (2)	
Eye	12 (2)	
Isolate	183 (27)	
Other	20 (3)	

*Values are no. (%) except as indicated. IQR, interquartile range. †Positive results/100,000 cultures. Fungal cultures identified by Logical Observation Identifier Names and Codes nos. 188243, 188573, 188805, 42804–5, and 580–1. ‡US Census, https://www2.census.gov/geo/pdfs/maps-data/maps/reference/us_regdiv.pdf.

**Figure:**
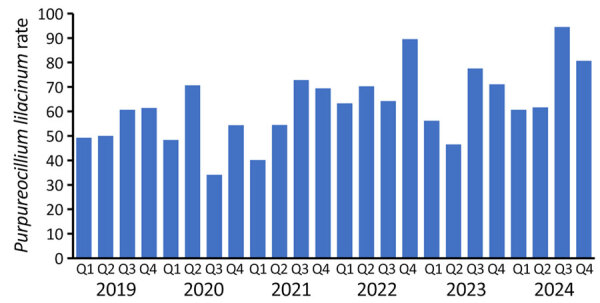
Rates of *Purpureocillium lilacinum* mold among laboratory culture results, United States. Graph shows *P. lilacinum* cultures per 100,000 fungal cultures by year and quarter during March 2019–February 2024.

Those commercial laboratory data suggest a recent increase in *P. lilacinum* cultures in the United States and a wide geographic distribution. Higher rates among male persons and older persons align with a previous study of *P. lilacinum* infections (*1*). The long time (*>*3 weeks) for culture growth and identification for this mold might lead to diagnostic delays and potentially inappropriate treatment (*1*,*4*).

The South Atlantic and Pacific Census divisions had substantially higher *P. lilacinum* culture rates than other regions of the United States. The high rates in the Pacific region could reflect a substantial uptick in cultures from that area during 2024–2025, including 60 cultures from a single facility that was the subject of a public health investigation (*6*). That event represented a pseudo-outbreak from environmental contamination rather than a true clinical outbreak, which is a rare occurrence. A previous *P. lilacinum* outbreak was linked to contaminated skin lotion (*7*). Two strains of *P. lilacinum* are used in the United States as agricultural bionematicides, PL251 registered in 2005 and PL11 registered in 2021 (*2*,*3*). Use of those bionematicides could possibly contribute to increased environmental presence and culture contamination during collection or in laboratories. Internationally, at least 1 case of subcutaneous *P. lilacinum* infection has been reported in association with bionematicide use (*8*). We could not assess environmental exposures in patients from whom the cultures in our study were derived; more data are needed on potential *P. lilacinum* environmental sources and their correlation with clinical culture positivity and infection risk.

Without clinical data, we were unable to determine patients’ underlying medical conditions and whether cultures represented true infection versus colonization. The most common specimen source was the respiratory tract, which can be colonized with various molds. Furthermore, nearly half of cultures were missing specimen type information, likely skewing our results; however, another study of confirmed infections found that skin was the most common infection site (*1*). Last, our data are a convenience sample and might not necessarily represent the entire US population.

In summary, mold infections generally are associated with substantially delayed and missed diagnoses (*9*). Further investigations are needed to understand the increased *P. lilacinum* culture rates, including examining bionematicide use, environmental changes, and clinical effects. Because *P. lilacinum* culture rates appear to be increasing, clinicians could consider nonculture-based diagnostic tools, such as microscopy (Appendix Figure 2), to help identify and confirm clinical infection earlier. 

Appendix Additional information on increased rates of *Purpureocillium lilacinum* mold among laboratory culture results, United States.

## References

[1] Sprute R, Salmanton-García J, Sal E, Malaj X, Ráčil Z, Ruiz de Alegría Puig C, et al.; FungiScope® ECMM/ISHAM Working Group. Invasive infections with Purpureocillium lilacinum: clinical characteristics and outcome of 101 cases from FungiScope® and the literature. J Antimicrob Chemother. 2021;76:1593–603. 10.1093/jac/dkab039 33599275 PMC8120338

[2] US Environmental Protection Agency. Registration decision for the new active ingredient *Purpureocillium lilacinum* strain PL11 [cited 2025 Apr 1]. https://www.regulations.gov/document/EPA-HQ-OPP-2016-0079-0013

[3] US Environmental Protection Agency. Paecilomyces species PC Codes 115002, 028826, and 115003 Interim registration review decision case number 6047 [cited 2025 Apr 1]. https://www.regulations.gov/document/EPA-HQ-OPP-2012-0403-0011

[4] Castelli MV, Alastruey-Izquierdo A, Cuesta I, Monzon A, Mellado E, Rodriguez-Tudela JL, et al. Susceptibility testing and molecular classification of *Paecilomyces* spp. Antimicrob Agents Chemother. 2008;52:2926–8. 10.1128/AAC.00538-08 18519716 PMC2493137

[5] Centers for Disease Control and Prevention. Reportable fungal diseases by state [cited 2025 Sep 12]. https://www.cdc.gov/fungal/php/case-reporting/index.html

[6] Centers for Disease Control and Prevention. Fungi faux pas: investigating a *Purpureocillium lilacinum* skin infection cluster—Washington, 2024 [cited 2025 Aug 20]. https://www.cdc.gov/eis-conference/php/media-resources/purpureocillium-lilacinum-skin-infection.html

[7] Orth B, Frei R, Itin PH, Rinaldi MG, Speck B, Gratwohl A, et al. Outbreak of invasive mycoses caused by *Paecilomyces lilacinus* from a contaminated skin lotion. Ann Intern Med. 1996;125:799–806. 10.7326/0003-4819-125-10-199611150-00003 8928986

[8] Zheng C, Li W, Gao Z, Yu Q, Yang L. Deep cutaneous fungal infection in an immunocompetent individual caused by a biological pesticide: a rare case report. BMC Infect Dis. 2025;25:341. 10.1186/s12879-025-10707-x 40069617 PMC11895385

[9] Caudron de Coquereaumont G, Couchepin J, Perentes JY, Krueger T, Lovis A, Rotman S, et al. Limited index of clinical suspicion and underdiagnosis of histopathologically documented invasive mold infections. Open Forum Infect Dis. 2021;8:ofab174. 10.1093/ofid/ofab174 PMC8446918 34549073

