# Supplementary material for: Increased Rates of Purpureocillium lilacinum Mold among Laboratory Culture Results, United States
Source: Emerg Infect Dis. 2025 Oct;31(10):2051–4. doi: 10.3201/eid3110.250715 (PMC12483104; doi:10.3201/eid3110.250715)
Supplement: Appendix — Additional information on increased rates of Purpureocillium lilacinum mold among laboratory culture results, United States. [file 25-0715-Techapp-s1.pdf]

Article DOI: https://doi.org/10.3201/eid3110.250715

EID cannot ensure accessibility for supplementary materials supplied by authors. Readers who have difficulty accessing supplementary content should contact the authors for assistance.

Increased Rates of *Purpureocillium lilacinum* among Laboratory Culture Results, United States

Appendix

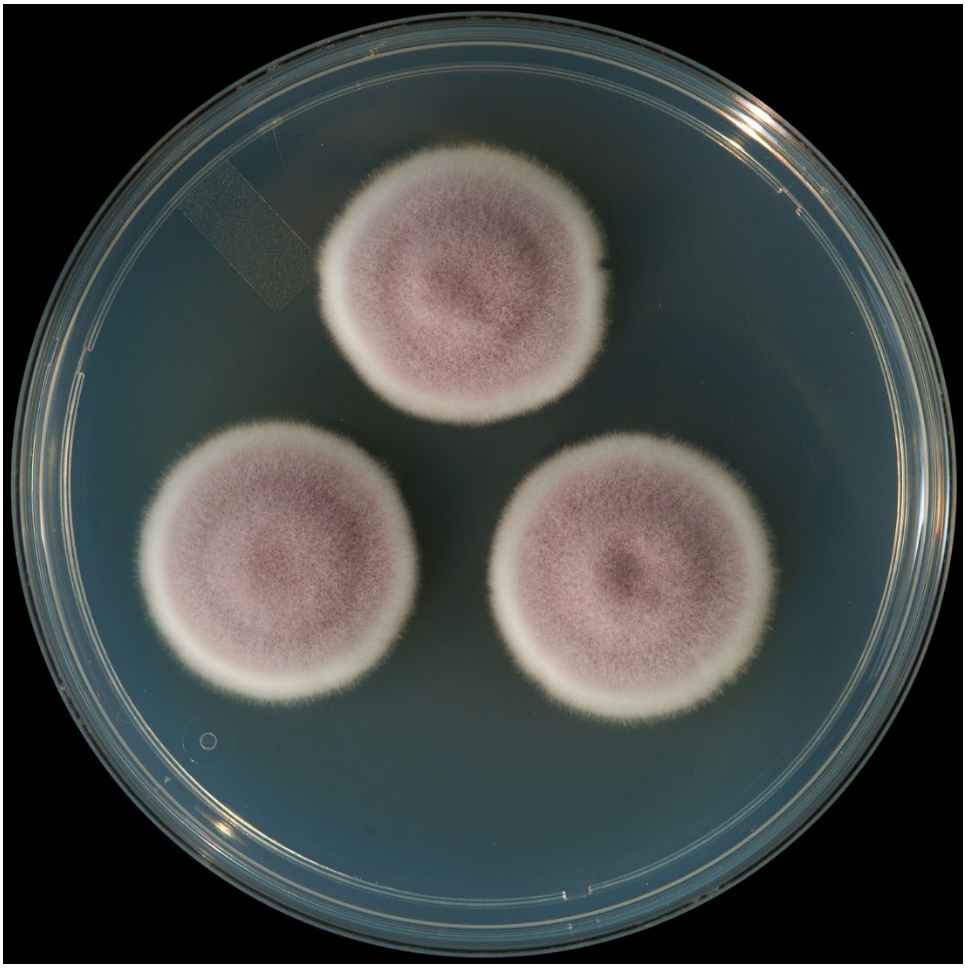

Appendix Figure 1. Photo of the front of a *Purpureocillium lilacinum* culture showing white-violet colony pigmentation. The colony color is the distinguishing feature of this species. Photo provided by the Mycotic Diseases Branch, Centers for Disease Control and Prevention. Photo credit to Jim Gaffeny, Centers for Disease Control and Prevention Photographic Services.

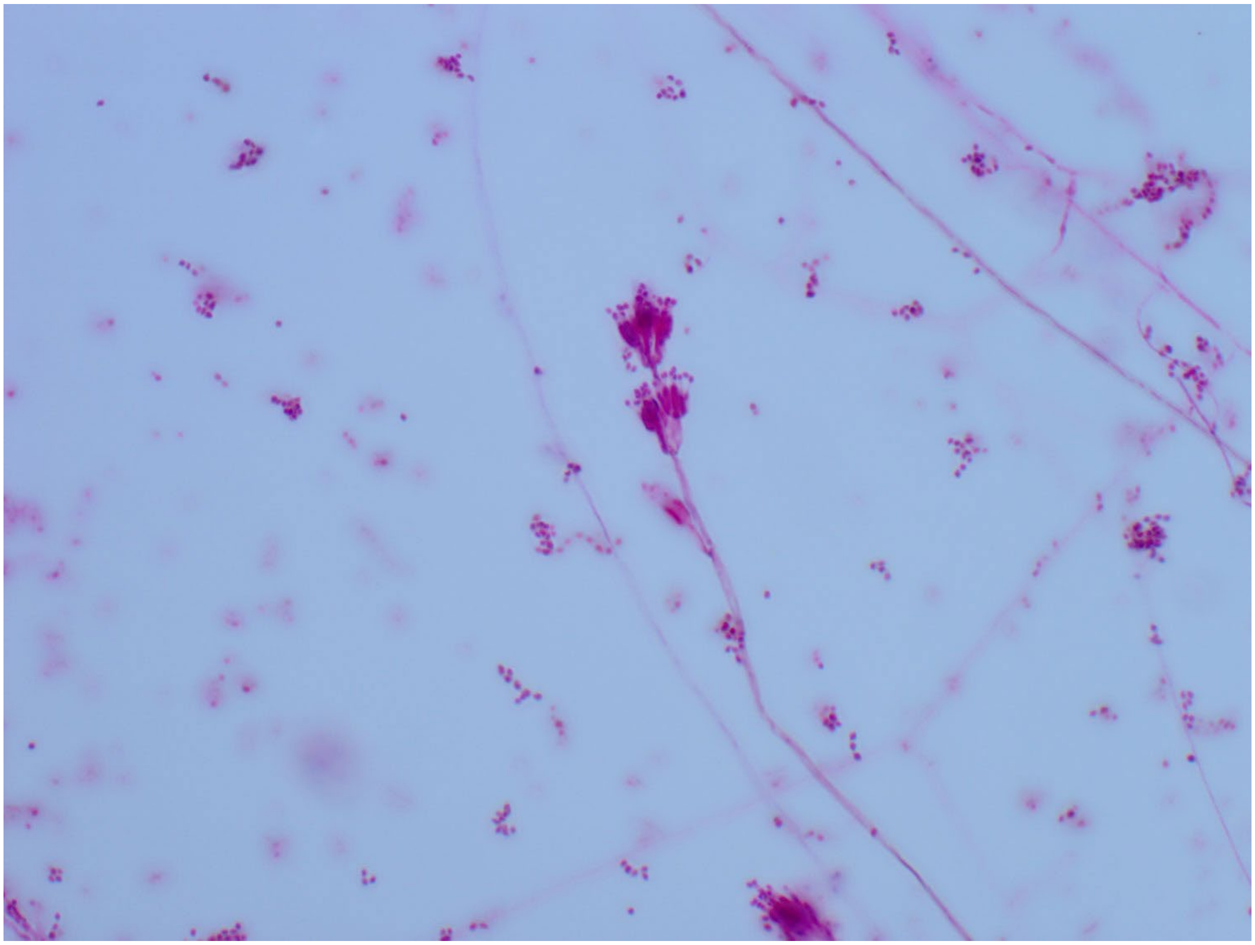

Appendix Figure 2. Photo of *Purpureocillium lilacinum* under microscopy at 40X magnification. *P. lilacinum* is difficult to distinguish microscopically from *Paecilomyces* spp. Microscopic key features include phialides with swollen bases that taper to a thin neck, rough-walled conidiophore stipes, and absence of chlamydospores. Conidia are ellipsoidal to fusiform. Photo provided by the Mycotic Diseases Branch, Centers for Disease Control and Prevention.
